# Draft Genome Sequences of *Acidithrix* sp. Strain C25 and *Acidocella* sp. Strain C78, Acidophiles Isolated from Iron-Rich Pelagic Aggregates (Iron Snow)

**DOI:** 10.1128/MRA.00102-21

**Published:** 2021-06-24

**Authors:** Qianqian Li, Rebecca E. Cooper, Carl-Eric Wegner, Shipeng Lu, Kirsten Küsel

**Affiliations:** a Institute of Biodiversity, Aquatic Geomicrobiology, Friedrich Schiller University Jena, Jena, Germany; b Institute of Botany, Jiangsu Province and Chinese Academy of Sciences, Nanjing, China; c The German Centre for Integrative Biodiversity Research (iDiv) Halle-Jena-Leipzig, Leipzig, Germany; University of Southern California

## Abstract

We report the draft genome sequences of two acidophiles, the Fe-oxidizing bacterium *Acidithrix* sp. strain C25 and the putative Fe-reducing *Acidocella* sp. strain C78. Both strains were isolated from iron-rich pelagic aggregates (iron snow) collected below the redoxcline at a 5-m depth in an acidic pit lake located in Germany (51°31′8.2″N, 13°41′34.7″E).

## ANNOUNCEMENT

Fe-cycling bacteria represent a large fraction of iron snow microbial communities in acidic pit lakes (1). *Acidithrix* sp. strain C25, a heterotrophic Fe(II)-oxidizing bacterium within the *Acidimicrobiaceae* family of the *Actinobacteria* phylum, is the second isolated and sequenced strain within the *Acidithrix* genus (2, 3). *Acidocella* sp. strain C78, a putative heterotrophic Fe(III)-reducing bacterium, belongs to the *Acetobacteraceae* family of the *Proteobacteria* phylum. *Acidocella* sp. strain YE4-N1-5-CH, isolated from this pit lake, and other *Acidocella* isolates have the capacity for dissimilatory Fe(III) reduction (4, 5). To further understand the metabolic potential and the contribution of these microbes to iron snow formation, we sequenced and analyzed these two genomes.

Both strains were isolated from 100 μl of diluted lake water (10^−1^ to 10^−3^) transferred to two types of overlay plates containing 25 mM FeSO_4_—iFeo (without a carbon source) for *Acidithrix* sp. C25 and YEo (0.2% yeast extract) for *Acidocella* sp. C78 (6, 7). Single colonies, transferred at least five times to establish pure cultures, were lysed and used for 16S rRNA gene PCR and phylogenetic analysis (7). To obtain sufficient biomass for DNA extraction, cultures were incubated in artificial pilot-plant water medium (APPW) amended with yeast extract (0.2 g liter^−1^). *Acidithrix* sp. C25 incubations were additionally amended with 25 mM FeSO_4_ (8). Genomic DNA was extracted using a phenol-chloroform-based protocol (9). Whole-genome sequencing was performed by RTL Genomics (Lubbock, TX, USA). Needle-sheared DNA was used to prepare 10- to 20-kb sequencing libraries using the PacBio SMRTbell template prep kit v1.0 without further size selection and was subsequently sequenced using P6-C4 chemistry with an 180-min collection protocol using a PacBio RS II platform (Pacific Biosciences, Menlo Park, CA) according to the standard manufacturer’s protocols. The raw reads were filtered and assembled *de novo* with the Hierarchical Genome Assembly Process v3 (HGAP3) (10) using default settings (https://github.com/ben-lerch/HGAP-3.0). Genome completeness and contamination were assessed with CheckM v1.0.13 using default parameters (11). Genome annotation was performed with RASTtk v2.0 using default parameters (12). The genome characteristics of both strains are listed in Table 1.

**TABLE 1:** Characteristics and accession numbers of draft genomes

Strain name	Genome size (bp)	Total PacBio sequences (bp)	PacBio sequence *N*_50_ (bp)	Coverage (×)	No. of contigs	G+C content (%)	*N*_50_ (bp)	Completeness (%)	Redundancy (%)	No. of genes	BioProject accession no.	Assembly accession no.
*Acidithrix* sp. C25	4,102,129	313,121,275	5,524	72	5	47.72	1,351,828	98.29	3.00	4,499	PRJEB40539	CAJHCI020000000.2
*Acidocella* sp. C78	3,250,836	145,349,460	3,896	38	59	67.54	96,816	94.53	1.00	3,219	PRJEB40546	CAJQZK010000000.1	

Analysis of the *Acidithrix* sp. C25 genome revealed genes encoding the complete Calvin-Benson-Bassham cycle, which indicates that the heterotrophic *Acidithrix* sp. C25 has the genetic potential for CO_2_ fixation. We could not identify homologs linked to Fe(II) oxidation under acidic conditions, such as *cyc2*, *cyt572*, *cyt579*, sulfocyanin, and *foxCD*. We found a gene encoding aromatic-l-amino-acid decarboxylase, which converts phenylalanine to phenethylamine, an aggregation-mediating infochemical within iron snow (7, 13).

The *Acidocella* sp. C78 genome encodes the complete Calvin-Benson-Bassham cycle, indicating the genetic capacity for CO_2_ fixation. Multiple genes encoding polysaccharide-degrading enzymes (e.g., glycosidase) and a gene encoding the methionine sulfoxide reductase heme-binding subunit, previously linked to Fe(III) reduction (14), were detected. These draft genome sequences are a valuable resource to understand iron snow functioning.

### Data availability.

The sequencing reads and assemblies of *Acidithrix* sp. C25 and *Acidocella* sp. C78 for this whole-genome sequencing project are available in ENA under the BioProject accession numbers PRJEB40539 and PRJEB40546, respectively, and the assembly accession numbers are listed in Table 1. The versions described in this paper are the first versions.
